# Entropy Engineering of 2D Materials

**DOI:** 10.1002/advs.202409404

**Published:** 2024-10-23

**Authors:** Hao Mei, Yuxuan Zhang, Panpan Zhang, Antonio Gaetano Ricciardulli, Paolo Samorì, Sheng Yang

**Affiliations:** ^1^ Frontiers Science Center for Transformative Molecules School of Chemistry and Chemical Engineering Shanghai Jiao Tong University Shanghai 200240 China; ^2^ State Key Laboratory of Material Processing and Die & Mould Technology School of Materials Science and Engineering Huazhong University of Science and Technology Wuhan 430074 China; ^3^ University of Strasbourg CNRS ISIS UMR 7006 Strasbourg 67000 France

**Keywords:** 2D materials, entropy engineering, high‐entropy materials, medium‐entropy materials

## Abstract

Entropy, a measure of disorder or uncertainty in the thermodynamics system, has been widely used to confer desirable functions to alloys and ceramics. The incorporation of three or more principal elements into a single sublattice increases the entropy to medium and high levels, imparting these materials a mélange of advanced mechanical and catalytic properties. In particular, when scaling down the dimensionality of crystals from bulk to the 2D space, the interplay between entropy stabilization and quantum confinement offers enticing opportunities for exploring new fundamental science and applications, since the structural ordering, phase stability, and local electronic states of these distorted 2D materials get significantly reshaped. During the last few years, the large family of high‐entropy 2D materials is rapidly expanding to host MXenes, hydrotalcites, chalcogenides, metal‐organic frameworks (MOFs), and many other uncharted members. Here, the recent advances in this dynamic field are reviewed, elucidating the influence of entropy on the fundamental properties and underlying elementary mechanisms of 2D materials. In particular, their structure‐property relationships resulting from theoretical predictions and experimental findings are discussed. Furthermore, an outlook on the key challenges and opportunities of such an emerging field of 2D materials is also provided.

## Graphical abstract

Entropy, a measure of disorder or uncertainty in thermodynamics systems, has been extensively employed to tailor the electrical, thermal, and optical properties of 2D materials. This review highlights recent progress in entropy engineering of 2D materials, covering synthesis, properties, and applications, especially the structure‐property relationships resulting from theoretical predictions and experimental findings. An outlook on the key challenges and opportunities of such an emerging field of 2D materials is also provided.

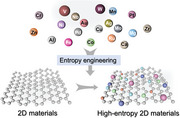

## 1 Introduction

In the course of human civilization, the fast‐paced progress in the synthesis of novel materials possessing unprecedented and diverse functions has been key to the emergence of new technologies responding to the needs of society. Toward this end, compositional and structural complexity has been exploited to design materials exhibiting specific properties for target applications.^[^ ^1^ ^]^ The early attempts, starting from the Bronze Age, took root in a “base element” paradigm, that is, by diluting one or rarely two base elements with traces of doping elements to improve the performance of composites. However, the accessible base elements were just a few, including copper, iron, or nickel, severely limiting the design space.

**Figure 1:**
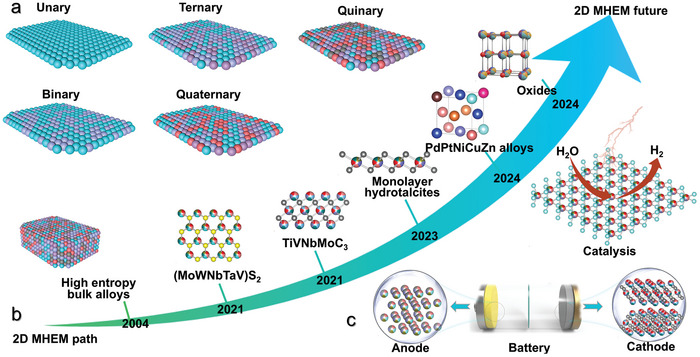
Schematic illustration of 2D MHEMs. a) Conceptual graphs from low entropy to high entropy. b) The development path of representative 2D MHEMs, such as MXene, sulfides， hydrotalcites and oxides. c) The energy storage and catalysis applications of 2D MHEMs.

Two decades ago, a different concept was proposed being focused on exploiting the previously unexplored structural configuration of multi‐element with high concentrations, which is in stark contrast to conventional trace doping strategy. In particular, this approach probes into the central region of the multi‐element phase diagram, not in the edges or corners.^[^ ^2^ ^]^ In these classes of compounds, entropy plays a crucial role as it can be used as a key parameter to tune the overall material thermodynamic, kinetic, and structural features.^[^ ^3^, ^4^, ^5^ ^]^ In particular, the combination of multiple elements of single‐phase crystal structures in equimolar or near‐equimolar quantity are merged to form a new class of materials defined by the number of constituting elements (n),^[^ ^6^ ^]^ named medium/high‐entropy materials (MHEMs), where medium and high refer to 3 ≤ n < 5 and n ≥ 5, respectively (**Figure** **1**a). Such a broad material family spans from metallic alloys to intermetallic and ceramic compounds, as well as microstructures with any number and any type of these phases.^[^ ^7^, ^8^, ^9^, ^10^ ^]^ Importantly, the modulation of entropy by integrating species with different atomic radii, electronegativity, ionization energy, etc., enables defectiveness, distortions, structural stabilization, and cocktail effects that lead to finely tunable properties in MHEMs,^[^ ^11^ ^]^ making them a promising versatile platform for a broad range of applications, including energy storage and conversion, optoelectronics, catalysis.^[^ ^12^ ^]^ In contrast to bulk MHEMs, in which entropy engineering represents an established tool for attaining in‐demand properties, its exploitation in the realm of 2D materials, mainly governed by single/two‐element structures, is still at a nascent stage.

2D materials are widely defined as sheet‐like nanomaterials characterized by a lateral size exceeding 100 nm and a thickness below 10 nm.^[^ ^13^ ^]^ Due to their atomic scale thickness, 2D materials boast a range of unique advantages, such as large surface area, superior mechanical strength, and ease of property modulation via surface engineering.^[^ ^14^ ^]^ Recently, efforts have been dedicated to the design of functional 2D materials through entropy engineering (i.e., 2D MHEMs). A series of MHEMs architectures down to the 2D limit have been developed so far, being based on transition metal chalcogenides (TMC), alloys, metal‐organic frameworks (MOF), MXenes, and hydrotalcites (Figure 1b,c).^[^ ^15^, ^16^, ^17^ ^]^ At such low dimensionality, due to quantum confinement, the medium/high‐entropy effect is expected to heavily affect the lattice framework and thus the properties of complex 2D materials. For instance, versatile electromagnetic properties, such as superconductive behavior, metal‐insulator transition, and antiferromagnetic transition, were detected in 2D high‐entropy (CoAu)_0.2_(RhIrPtPd)_0.8_Te_2_, (TiVCrNbTa)S_2_ and (MnFeCoNi)PS_3_.^[^ ^18^ ^]^ Thus, the integration of medium/high‐entropy concepts and 2D structures holds the promise of creating a versatile library of materials with attractive physicochemical properties.

Previous review articles mainly discussed the theoretical property prediction of high‐entropy 2D materials or potential applications of high‐entropy van der Waals (vdW) materials.^[^ ^19^, ^20^ ^]^ However, some key elements of 2D high‐entropy materials, such as entropy engineering principles, crystal structural aberrations, and unique physicochemical properties, have not yet been reviewed in‐depth. Herein, we comprehensively review the entropy engineering of 2D materials, including basic definitions, general materials categories, fundamental synthesis mechanisms, unique properties, and representative applications. Furthermore, the entropy stabilization effect, disordered multiple atomic distribution, and local electronic structure in 2D systems are thoroughly discussed. We also critically assess the advantages and weaknesses of 2D MHEMs, providing an outlook on the key challenges and opportunities.

## 2 Theoretical Concept and Core Effects of 2D MHEMs

### 2.1 Thermodynamic Terminology

**Figure 2:**
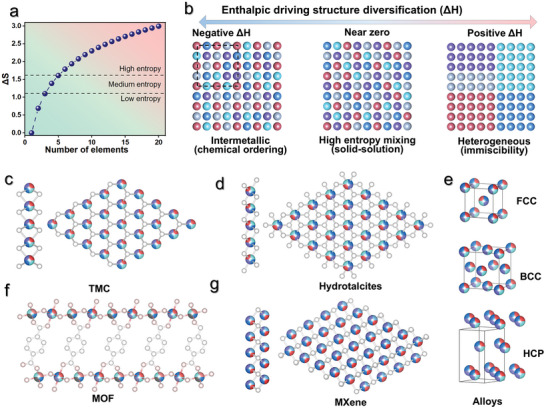
a) The configurational entropy under different numbers of elements, which is calculated based on Equation (3). b) Effect of enthalpy value on phase structure formation mechanism. c–g) Crystal structure of current reported 2D MHEMs, including TMC, MOF, MXene, hydrotalcites, and alloys with face‐centered cubic (FCC), body‐centered cubic (BCC), and hexagonal‐close packed (HCP) structure.

To avoid confusion and inconsistencies, choosing the right term for medium and high entropy for a wide set of materials is critical. In theory, the mixed entropy of a multi‐component system consists of four fractions, as shown below:^[^ ^21^ ^]^

(1) $$\Delta S_{\mathrm{mix}}=\Delta S_{\mathrm{conf}}+\Delta S_{\mathrm{vib}}+\Delta S_{\mathrm{mag}}+\Delta S_{\mathrm{elec}}\;$$

where ΔS_conf_, ΔS_vib_, ΔS_mag,_ and ΔS_elec_ are configurational entropy, vibrational entropy, magnetic dipole entropy, and electronic randomness entropy, respectively. Typically, configurational entropy attracts more dominant attention than its counterparts, which directly affects phase stability. However, to accurately determine the total entropy of a multi‐component system, it is essential to account for contributions beyond ΔS_conf_, such as ΔS_vib_, ΔS_mag,_ and ΔS_elec_. For instance, in the case of ΔS_vib_, previous studies using finite‐temperature ab initio methods have shown that while vibrational entropy can notably affect stability, its absolute magnitude does not always directly correlate with relative phase stabilities. Instead, the variation in vibrational entropy between different phases is more pertinent. Furthermore, it has been observed that vibrational entropy may decrease with increasing element diversity or number, complicating the prediction of its impact on phase stability. It should be noted that these considerations have been done mainly on alloys. Nevertheless, as the library of 2D MHEMs is broad and diverse, general calculations on vibrational entropy, magnetic dipole entropy, and electronic randomness entropy contribution are inherently challenging. Therefore, despite the role of the different entropy components, S_conf_ can be considered as the key factor. In a typical 2D MHEMs composed of metal and non‐metal species, ΔS_conf_ can be written as the following equation:^[^ ^6^ ^]^

(2) $$\Delta S_{\mathrm{conf}}\;=-R\left[{\left(\sum_{1}^{n}X_{i}\ln X_{i}\right)}_{\mathrm{metal}}+{\left(\sum_{1}^{m}X_{j}\ln X_{j}\right)}_{\mathrm{non}-\mathrm{metal}}\right]\;$$

where *R*, *X_i_* and *X_j_* are the ideal gas constant, and molar content of metal and non‐metal species, respectively. Obviously, the value of ΔS_conf_ increases with the element number. For traditional multi‐components alloys with equimolar, X = 1/*n*, this Equation (2) can be simplified:

(3) $$\Delta S_{\mathrm{conf}}\;=\;R\ln n\;\;$$

with *n* as the number of elements. Based on theoretical calculations, at the equimolar ratio, the ΔS of unary, binary, ternary, quaternary, and quinary alloys are 0, 0.69, 1.10, 1.39, and 1.61R, respectively. Thus, a number of researches have recommended a quantitative value of configurational entropy to distinguish medium (1.10–1.61R) and high entropy (≥1.61R), as shown in **Figure** **2**a. Alternatively, MHEMs can be also defined by their compositions with disordered distribution, such as numbers of metal elements in equimolar or near‐equimolar content (i.e., 3–4 and 5 or more metal elements represent medium entropy and high entropy, respectively).

In thermodynamic theory, the formation of 2D MHEMs is related to the interplay between entropy and enthalpy, expressed as the Gibbs free energy equation:

(4) $$▵G_{\mathrm{mix}}=▵H_{\mathrm{mix}}-T▵S_{\mathrm{mix}}\;$$

where T and △H_mix_ are temperature and enthalpy changes in mixing multiple elements. The interplay between △H_mix_ and ‐T△S_mix_, which are usually positive and negative, respectively, determines the resulting phase structure. On the one hand, the ‐T△S_mix_ depends on the number of elements and temperature, simultaneously serving as the driving force to achieve single phase mixing, which is well‐known as the “entropy stabilization effect”.^[^ ^22^ ^]^ On the other hand, enthalpy primarily hinging on properties of constituent elements, also directly influences the phase structure under near‐equilibrium conditions.^[^ ^11^ ^]^ As shown in Figure 2b, the intermetallic compounds with highly ordered element distribution are favored with a negative value of △H_mix_. In contrast, at positive △H_mix_ values, phase separation occurs, yielding heterogeneous structures. It is worth noting that for the near‐zero value of △H_mix_, a mixture of medium and high entropy is generally obtained. Furthermore, temperature is also a crucial physical parameter, which significantly influences the element distribution and crystal structure. In this regard, a detailed discussion will be provided in the section on synthetic methods.

### 2.2 Core Effects of 2D MHEMs

Entropy theory has been extensively employed for the design and synthesis of diverse functional 2D MHEMs (Figure 2c–g), including transition metal chalcogenides and halides, MOFs, MXenes, and hydrotalcites. To date, it has been discovered that the produced 2D MHEMs inherit core effects of traditional bulk forms, such as sluggish‐diffusion effects, drastic‐lattice distortion, and cocktail effects.^[^ ^23^ ^]^ Besides, as MHEMs thickness approaches the single‐unit‐cell limit, the possibility of discovering unusual quantum phenomena and exotic chemical properties is greatly enhanced due to quantum‐confinement effects, strongly influencing the material properties. The drastic‐lattice‐distortion effects directly alter the structural properties and shape diverse defects like point, line, and interface defects.^[^ ^24^ ^]^ When coupled with atomic‐thickness effects, these structural aberrations are further amplified, leading to strong surface lattice strain of 2D MHEMs. The sluggish‐diffusion effects reduce internal atomic migration and similarly decelerate phase transformations.^[^ ^25^ ^]^ The cocktail effects denote a form of synergistic outcome arising from the interaction among diverse elements.^[^ ^26^ ^]^ A detailed description of these effects and their impacts on the properties of the material are provided in the following sections.

#### 2.2.1 Drastic‐Lattice‐Distortion Effects

MHEMs require the integration of diverse elements into a single‐phase lattice structure. Once elements with different atomic radius, chemical valences, and electronegativities coexist in a shared sublattice, the lattice distortion and strain emerge thereby altering the overall properties of MHEMs.^[^ ^27^ ^]^ Generally, the lattice distortion and strain increase as the disparity among elements widens. While the lattice strain of the localized lattice area may be minor, the cumulative effect on the long‐range scope is substantial and consequential. For instance, research on alloys has shown that a 1% interatomic spacing change is likely to cause approximately a 12% variation in interatomic force.^[^ ^28^ ^]^ To date, the prevalent depiction of lattice distortion usually focuses on the atomic radius differences (δ), which can be directly evaluated by the following formula:^[^ ^29^ ^]^

(5) $$\delta =100\sqrt{\sum_{i}^{n}C_{i}{\left(1-\frac{r_{i}}{\bar{r}}\right)}^{2}},\;\;\bar{r}=\sum_{i=1}^{n}c_{i}r_{i}\;\;$$

where *n*, *r*, and *c_i_* are the number of elements, atomic radius, and concentration of constituent element i, respectively. Several studies have shown that this parameter is valid for estimating phase stability of high‐entropy materials.^[^ ^30^ ^]^ When scaling down the crystals from bulk to the 2D space, the lattice distortion may be further strengthened. Therefore, a number of structural defects, such as vacancies, grain boundaries, and dislocations have been discovered in 2D MHEMs.^[^ ^23^ ^]^

#### 2.2.2 Atomic‐Thickness Effects

The atomic‐thickness effects impart many unique characteristics to MHEMs that are different from typical bulk forms. First, the phonon/charge carriers of 2D MHEMs are confined to the atomic‐thickness region, which is favorable for the development of ultrathin yet sensitive optoelectronic devices.^[^ ^31^ ^]^ Second, considering the strong lattice distortion limited at 2D scale, atomic strain and surface defects are highly possible. Third, the strong in‐plane covalent bond and weak interlayer forces lead to substantial flexibility and mechanical strength, which make MHEMs promising flexible wearable electronics devices.^[^ ^32^ ^]^ Last but not least, atomic thickness endows materials with a large accessible surface area, which further enables high tunability of properties and functionalities via ingenious design by means of defect/phase/strain engineering.^[^ ^33^ ^]^ These remarkable characteristics offer advantages for applications propelled by surface chemical reactions like electrocatalysis, batteries, and sensing.

#### 2.2.3 Cocktail Effects

The concept of cocktail effects, initially introduced by Ranganathan to depict amorphous alloys, gum metals, and high‐entropy alloys, suggests that blending various elemental species may result in unexpectedly remarkable properties.^[^ ^34^ ^]^ In 2D MHEMs, the cocktail effect refers to the interaction of multiple constituent elements in MHEMs, resulting in superior performance.^[^ ^35^ ^]^ It emphasizes that the synergistic working effect dominates the properties of MHEMs rather than solely contribution from any of the specific elements. The interplay between diverse components can be significantly altered by the element types and stoichiometry, which further affect the material's properties. Additionally, it is highly feasible to achieve outstanding performance in MHEMs by replacing scarce and/or toxic elements with abundant and sustainable ones, thus contributing to sustainability.

#### 2.2.4 Sluggish‐Diffusion Effects

The sluggish‐diffusion effect of 2D MHEMs refers to the migration of atoms in severely disordered lattices is limited. In general, MHEMs exhibit varying local energy in the sublattice area, attributed to various bonding types of constitute elements. Compared to the almost same diffusion barrier in conventional materials, the energy barrier along the atom diffusion pathway for MHEMs is continuous. Thus, during the internal migration of atoms, a high probability of falling into high‐barrier sites occurs, named the “trapping effect”, significantly slowing down the diffusion rate.^[^ ^12^ ^]^ Furthermore, the sluggish atomic diffusion also influences the phase transformation rate. Recent studies have demonstrated that sluggish‐diffusion effects stabilize the microstructure of MHEMs and alter their performance in applications. For instance, the dynamic stability of a 2D high‐entropy electrocatalyst would be strengthened through sluggish‐diffusion effects, enabling excellent catalytic performance even in harsh environments for extended periods.^[^ ^36^ ^]^

## 3 Synthesis of 2D MHEMs

### 3.1 General Principles for Making 2D MHEMs

The controlled preparation of high‐quality 2D MHEMs with desired composition, phase purity, and structure is key for practical applications. However, increasing the entropy of materials while retaining their 2D morphology represents the main challenge in the synthesis of this emerging class of low‐dimensional materials. In terms of structural assembly, the typical synthesis mainly relies on bottom‐up and top‐down approaches. Bottom‐up strategies rely on the controlled assembly of small molecules forming macroscale 2D frameworks. In contrast, top‐down methods are generally based on the exfoliation of bulk materials using chemical, physical, and mechanical techniques. For the sake of entropy engineering of 2D materials, overcoming the intrinsic immiscibility of metal atoms driven by atomic radius difference, electronegativity disparity, and valence electron concentration is the most critical point. Representative examples of 2D MHEMs produced based on increasing the entropy of the host phase, including alloys, MXene, hydrotalcites, and metal sulfides are summarized in **Table** **1**.^[^ ^37^, ^38^, ^39^, ^40^, ^41^, ^42^, ^43^, ^44^ ^]^ The inherent properties of elements, including electronegativity, atomic/ionic radius, and electronic structure, should be carefully considered in designing the synthesis methods.

**Table 1:** Representative examples of the reported 2D MHEMs.

MHEMs	Number of metallic elements	Metal contents [at. %]	Entropy Level	Ref
Alloys	3	43%Pd, 28%Pt, 29%Ag	medium	[37]
Alloys	5	23%Pt, 20%Pd, 17%Ir, 16%Ru, 24%Ag	high	[38]
Alloys	5	22%Fe, 22%Ni, 22%Co, 22%Cr,11%Nb	high	[39]
MXene	5	7.1%Ti, 8.6%Nb, 6.6%Ta, 0.1%Cr, 4.9%V	high	[40]
MXene	3	33.3%Ti, 15% V, 1.7%Cr	medium	[41]
Hydrotalcites	5	3.31%Mn, 7.88%Fe, 4.16%Co, 8.54%Ni, 7.38%Cu	high	[42]
TMC	5	12%Co, 2% V, 2%Ni, 2%Mn, 2%Zn	high	[43]
TMC	5	12% Ru, 6%Ir, 8%Fe, 2%Co, 2%Ni	high	[44]

### 3.2 Methods to Increase the Entropy of 2D Materials

**Figure 3:**
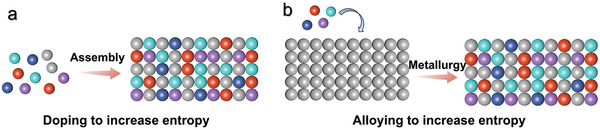
a) Doping strategy to increase the entropy of 2D materials. b) Alloying reaction for achieving medium/high entropy.

The designing methods to increase the entropy of 2D materials are usually based on doping and alloying strategies. The doping strategy involves directly mixing various metal precursors to acquire a medium/high‐entropy structure (**Figure** **3**a). Generally, oriented by a 2D lattice framework, all metal precursors totally dissolve and dope into to targeted lattice structure under extreme conditions. Alternatively, alloying by infiltrating other components into the target 2D monocomponent template represents another effective way to produce 2D MHEMs, as shown in Figure 3b. According to Gibb's free energy formula (Equation (4)), in addition to △S_mix_ and △H_mix_, doping/alloying temperature constitutes a pivotal parameter, regulating the feasibility of medium/high‐entropy systems. Ultrahigh doping/alloying temperatures and ultrafast heating rates were generally employed to achieve a stable homogeneously mixed solid‐solution state, as the blending of metals with divergent characteristics is facilitated. For instance, the classical carbothermal shock synthesis was even conducted at 2000 K ultrahigh temperature and 10^5^ K/s ultrafast heating rate.^[^ ^45^ ^]^ However, such extreme conditions do not allow structural control over the production of MHEMs in a 2D fashion. Thus, the currently reported synthetic methods to increase the entropy of 2D materials, such as wet‐chemical synthesis, chemical vapor deposition (CVD) doping, and calcination alloying, are usually carried out under relatively mild conditions. In the following section, state‐of‐the‐art synthesis methods will be introduced, with a focus on the impact of experimental parameters on the features including surface vacancies, lateral size, thickness, and phase structure of the materials.

#### 3.2.1 Wet‐Chemical Synthesis

**Figure 4:**
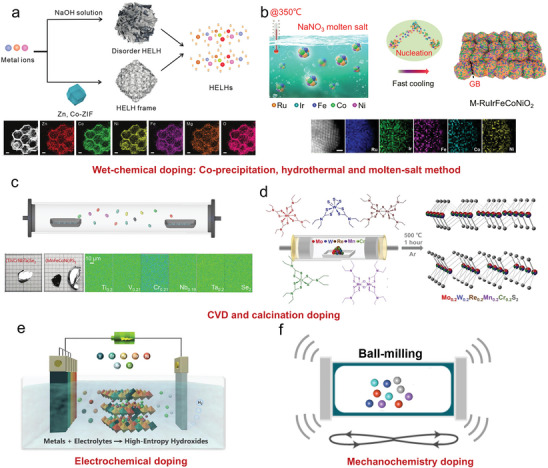
Doping synthesis to increase the entropy of 2D materials, including wet‐chemical doping, chemical vapor deposition doping, electrochemical doping, and mechanochemistry doping. a) Wet‐chemical etching of Zn/Co‐MOF to prepare high‐entropy hydrotalcites. Reproduced with permission.^[^ ^52^ ^]^ Copyright 2023, Wiley‐VCH. b) Molten‐salt synthesis of 2D high‐entropy RuIrFeCoNiO_2_. Reproduced under the terms of the CC‐BY‐NC licence.^[^ ^44^ ^]^ Copyright 2023, AAAS. c) CVD synthesis of MHEMs monocrystal, including Metal dichalcogenides, halides, and phosphorus trisulfides. Reproduced with permission.^[^ ^18^ ^]^ Copyright 2021, American Chemical Society. d) Calcination synthesis of 2D high‐entropy Mo_0.2_W_0.2_Re_0.2_Mn_0.2_Cr_0.2_S_2_. Reproduced with permission.^[^ ^55^ ^]^ Copyright 2023, Wiley‐VCH. e) Direct electrodeposition synthesis of high‐entropy hydrotalcites. Reproduced with permission.^[^ ^57^ ^]^ Copyright 2022, Elsevier. f) Mechanochemistry doping synthesis.

Wet‐chemical synthesis, including co‐precipitation, solvothermal, and molten‐salt reactions (**Figure** **4**a,b), is the most frequently‐used strategy to produce 2D MHEMs.^[^ ^46^ *^,^* ^47^ ^]^ In order to promote co‐precipitation and solvothermal process, a stoichiometric ratio of various metal salts is dissolved in a specific solvent to form a mixture. Then the homogeneous solution is transferred into an autoclave to complete the synthesis. Generally, the synthesis of 2D MHEMs is based on a typical crystallization mechanism, which involves crystal nucleation and subsequent growth.^[^ ^46^ ^]^ The crystal nucleation is generated by the spontaneous random aggregation of ions at a supersaturating state. On the basis of thermodynamics theory, this process needs to cross the free energy barrier affected by the reaction temperature. Based on LaMer theory, the fast nucleation is conductive to reach a single‐phase solid solution structure and higher entropy.^[^ ^48^ ^]^ On the contrary, the slower nucleation may lead to phase separation and decreased configurational entropy. The subsequent growth has important effects on the resulting crystallinity, morphology, and size, which can be regulated by surfactants. A variety of 2D medium/high‐entropy hydrotalcites are synthesized by co‐precipitation or solvothermal doping. The hydrotalcite crystal structure presents sufficient flexibility to endure the mismatching of metal ion radius and coordination states. Considering the 133 pm radius of hydroxyl ion, the metal ion radius is in the range of 55 to 88 pm, to ensure the octahedral structure stability of metal hydroxides.^[^ ^49^ ^]^ Currently, a maximum of nine metal elements can co‐exist in the hydroxide structure framework, and the obtained novenary hydroxide still presents ultrathin thickness. Analogously, the multiple metal ions can also co‐exist in MOF.^[^ ^50^ ^]^ Previous works have demonstrated the conformal conversion of MOF into hydroxides, oxides, and sulfides.^[^ ^51^ ^]^ With this respect, Tang et al. reported the preparation of high‐entropy hydrotalcites via etching Zn/Co‐ZIF in a polymetallic mixing solution (Figure 4a).^[^ ^52^ ^]^ The prepared high‐entropy hydrotalcite presented 1.07 nm thickness, hollow frame structure, and amorphous features.

Compared to co‐precipitation or solvothermal methods, molten salt‐assisted synthesis is carried out at a higher temperature and heating rate. Thus, the guest metal ions are more easily doped into the host phase structure to achieve medium/high‐entropy mixing. For instance, Huang et al. reported the introduction of Ni/Co/Fe/Ir ions into RuO_2_ crystal structure under NaNO_3_ molten salt and successfully produced 2D high‐entropy (FeCoNiIrRu)O_2_ with 3 nm thickness.^[^ ^44^ ^]^ The synthesis was carried out under rapid high‐temperature mixing and fast cooling, the high‐entropy (FeCoNiIrRu)O_2_ still presents many defects induced by lattice‐distortion effects, such as vacancies and grain boundaries. In addition to hydroxides and oxides, a large class of Pd‐based alloys with face‐centered cubic structures can be synthesized through the reduction of metal salts in wet‐chemical conditions.^[^ ^53^ ^]^ However, these Pd‐based alloys produced by direct co‐reduction synthesis all present quasi‐2D features, whose lateral size is in the range of 50–200 nm. In general, the wet‐chemical doping synthesis is currently applicable to limited materials such as hydrotalcites, oxides, and Pd‐based alloys.

#### 3.2.2 CVD and Calcination Doping

Calcination and CVD doping are alternative effective synthetic techniques (Figure 4c,d), which are mainly performed for making high‐entropy MAX phases and TMC. CVD is a conventional synthetic technique that uses a carrier gas at high temperatures to yield highly crystalline materials at the interface with a substrate. In contrast, calcination is a process that occurs through solid or liquid reactions to assemble species with high‐entropy mixing. Many experimental parameters, such as substrate interface, heating rate, reaction time, cooling rate, and gas atmosphere, have great impacts on the calcination process and final products. Considering the higher reaction temperature and ramping rate, various metal ions are more likely to integrate with ordered crystal domains rather than in a random fashion through wet‐chemical doping. The typical calcination process was often employed for the preparation of MAX phases by solid phase reactions. Huang et al. reported the synthesis of medium/high‐entropy M_2_SnC (M = Ti/V/Nb/Zr/Hf) MAX phases using metal powder and carbon as starting reagents.^[^ ^54^ ^]^ In order to overcome the incompatibility between the multiple components, the whole process was conducted at 1350 °C under 30 MPa. Besides, using metal dithiocarbamate as a precursor, Lewis et al. performed the rapid synthesis of high‐entropy Mo_0.2_W_0.2_Re_0.2_Mn_0.2_Cr_0.2_S_2_ nanosheets via calcination process (Figure 4d).^[^ ^55^ ^]^ Different from calcination, CVD represents another effective method to yield high‐quality single‐crystal 2D materials. For example, Hosono et al. synthesized a series of high‐entropy layered materials via CVD doping, including mixed metal dichalcogenides, halides, and phosphorus trisulfides, which greatly expanded the library of 2D MHEMs (Figure 4c).^[^ ^18^ ^]^ The chemical/physical exfoliation process was essential to exfoliate MAX crystal or bulk TMC into 2D sheets. In general, the chemical etching of MAX performed in HF or F‐containing etching solution led to mixed surface terminations including –F, ‐O, and –OH.^[^ ^56^ ^]^ Unlike the chemical etching process of MAX, the weak interlayer interaction of TMC can be eliminated by mechanical force, such as the scotchtape and ball‐milling methods.

#### 3.2.3 Electrodeposition Doping

Electrodeposition is a cost‐effective and scalable synthetic method. As shown in Figure 4e, the electrodeposition system includes an electrochemical workstation, an electrolytic tank, the electrolyte, and electrodes. The reactants in the electrolyte are oxidized or reduced upon the application of a potential, and continuously deposited on the working electrode. Therefore, the crystal nucleation and subsequent growth of target materials can be controlled by changing the electrodeposition mode (DC/pulse), overpotential, solution viscosity, and stirring speed, thus obtaining different film morphologies and deposition sizes. The redox potential of various metal ions is a key to increasing the entropy of materials via electrodeposition doping. In general, the high potential will promote fast deposition of metal ions onto the electrode surface, achieving a high‐entropy mixing. In this regard, Dong and coworkers developed a universal and low‐cost solution electrodeposition approach for producing high‐entropy layered CoFeVCrNi‐LDH (Figure 4e).^[^ ^57^ ^]^ The whole process was conducted in a two‐electrode system using metal foil (Co/Fe/V/Cr/Ni) and Pt plate, as anode and cathode, respectively. A series of 2D MHEMs were produced, including ternary CoFeNi‐LDH, pentabasic CoFeNiMnCr‐LDH, senary CoFeNiZnCrAl‐LDH, octonary CoFeNiZnCrAlCuMn‐LDH, demonstrating the general applicability of such electrochemical synthesis. Alternatively, the work from Reza et al. provided further evidence of high‐yield synthesis of high‐entropy (FeMnNiCaMg)_x_OH_y_ at a high current density of 232 mA/cm^2^.^[^ ^58^ ^]^

#### 3.2.4 Mechanochemistry Doping

Mechanochemistry doping refers to the use of mechanical energy to trigger chemical reactions, such as the ball‐milling technique (Figure 4f). Generally, guest metal species are introduced into a host crystal structure under high mechanical force, resulting in a high‐entropy state. Recently, due to the recent progress in the modernization of mechanical equipment, mechanochemical synthesis with solvent‐free and eco‐friendly attributes is rapidly emerging as a promising alternative for the scalable production of MHEMs. Nevertheless, to overcome the immiscibility of diverse elements and trigger their mixing, mechanochemical synthesis should be coupled with other methods, such as heat and optical radiation. Dai et al. performed a class of mild mechanochemical coupled with calcination reactions for preparing high‐entropy K_1–_ *_x_* Na*_x_* (MgMnFeCoNi)F_3_ perovskite fluorides and (CeZrHfTiLa)O_x_ fluorite oxides.^[^ ^59^ ^]^ Likewise, Li et al. synthesized medium‐entropy NiZrCu‐H_2_BDC‐MOF with an average thickness of 4 nm via ultrasonic‐assisted approaches.^[^ ^60^ ^]^

#### 3.2.5 Atomic Exchange Alloying

**Figure 5:**
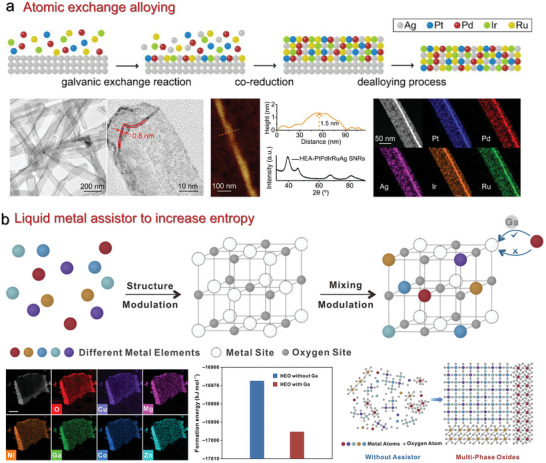
a) Atomic exchange alloying reactions to increase entropy. The high‐entropy AgPtPdIrRu nanoribbon was synthesized based on the Ag template. Reproduced with permission.^[^ ^38^ ^]^ Copyright 2022, American Chemical Society. b) Preparation of 2D high‐entropy oxides by doping multifarious metal precursors with liquid Ga assistor. Clearly, the Ga assistor reduced the formation energy of high‐entropy oxides and inhibited phase separation. Reproduced with permission.^[^ ^63^ ^]^ Copyright 2024, American Chemical Society.

Alloying or metallurgy is a classic approach to increasing the entropy of 2D materials. In this process, the initial metal template reacts with other metal sources due to the difference in reduction potential. For instance, Guo et al. introduced a universal synthesis technique to prepare high‐entropy alloys on an Ag nanowires‐based template (**Figure** **5**a).^[^ ^38^ ^]^ The formation process of high‐entropy alloy is composed of three steps: 1) initial nucleation via galvanic ion exchange process induced by the reduction potential difference; 2) subsequent crystal growth by continuous co‐reduction of metal sources; 3) dealloying process by nitric acid. In contrast to high‐temperature metallurgy, the galvanic ion exchange process was carried out in the liquid phase at 200 °C. Furthermore, senary PtPdIrRuAuAg, septenary PtPdIrRuAuRhAg, and octonary PtPdIrRuAuRhOsAg nanoribbons with 1.5 nm thickness, 50–150 nm width, and micronmeter‐scale length, were successfully synthesized. Analogously, Skrabalak et al. reported the alloying process for the preparation of single‐phase high‐entropy alloys (Pd, Sn, Ag, Co Cu, Pt, Ni, and Au components). Initially, bimetal PdCu and AuCu alloys were employed as fundamental cores and coated by other shell metals (Pt, Ni, Co et al.) via a co‐reduction process.^[^ ^61^ ^]^ After subsequent annealing, the core‐shell metal precursor was transformed into high‐entropy alloys due to internal galvanic exchange reactions. Noteworthy, the relative redox potentials (E_red_) of metal determine internal galvanic exchange reactions. Thus, the metal with high E_red_ tended to replace the one with lower E_red_.

#### 3.2.6 Other Synthetic Methods

Many other synthetic approaches including magnetron sputtering and liquid metal assisted synthesis have recently emerged to prepare 2D MHEMs. Yang et al. introduced advanced magnetron sputtering to yield 2D metals on modified polyvinyl alcohol substrates and demonstrated the general synthesis of Ti, medium‐entropy ZrCuAlNi alloys, high‐entropy FeCoNiCrNb alloys.^[^ ^39^ ^]^ Noteworthy, the obtained 2D medium/high‐entropy alloys were endowed with amorphous characteristics, micrometer‐scale lateral size, and thicknesses down to a few tens of nanometers. The thickness, crystallinity, and components of 2D alloy film can be effectively controlled by regulating several magnetron sputtering parameters, such as sputtering power and gas flow rate. Besides magnetron sputtering, Fu et al. found liquid Ga could assist in overcoming the elemental immiscibility, enabling the production of a broad variety of high‐entropy alloys in the form of nanoparticles under relatively mild conditions.^[^ ^62^ ^]^ In recent work, they showed the possibility of extending such a synthetic strategy to yield a series of 2D high‐entropy oxides with phase controllability, including rock‐salt, spinel, perovskite, and fluorite structures, as shown in Figure 5b.^[^ ^63^ ^]^ According to density functional theory calculations (DFT), liquid Ga reduced the formation energy of high‐entropy oxides, inhibiting phase separation. From a broader perspective, liquid metal might be used to extend the family of high‐quality 2D MHEM, such as carbides and sulfides.

#### 3.2.7 Comparison of Various Synthesis

The controllable preparation of high‐quality 2D MHEMs with desired composition, phase structure, and morphology is of great importance for the further development of the field. Wet‐chemical methods are widely used for synthesizing a variety of 2D MHEMs, including hydrotalcites, oxides, and sulfides. By designing experimental parameters such as reaction temperature, time, and solvent systems, the nanosheet size and phase structure can be effectively controlled. In general, wet‐chemical synthesis is performed in mild conditions and does not require sophisticated equipment. Nonetheless, current approaches are affected by low yield and lack of controlled atomic distribution. In contrast, CVD and calcination strategies can be used to form high‐quality 2D MHEMs with excellent phase purity and tunable morphology. However, this method requires advanced equipment and high energy consumption. As a result, upscaling is severely hindered. Besides, mechanochemical methods, electrodeposition and magnetron sputtering have been reported for synthesizing 2D MHEMs. Nevertheless, these methods are still in their infancy. A major challenge in mechanochemical synthesis is obtaining high‐quality 2D sheets with controlled thickness and size. So far, electrodeposition and magnetron sputtering are still limited to the synthesis of a limited class of materials, such as hydrotalcite or alloys. In perspective, further efforts are needed to enhance the intermixing of components while keeping a 2D architecture.

## 4 2D MHEMs Structure and Properties

Up to now, numerous 2D MHEMs have been produced via multifarious advanced synthetic technologies. Despite distinct differences in components, chemical bonding, and phase structures, the family of 2D MHEMs can be classified into layered and non‐layered architectures. The typical examples of 2D layered MHEMs are MXene and hydrotalcite, which possess strong in‐plane covalent bonds and weak interlayer Van der Waals (vdW) forces. On the contrary, although a number of MHEMs are endowed with non‐layered structures, their atomically thin analogues can be synthesized by bottom‐up approaches, such as Pd‐based metallene and metal oxides. In this section, we will discuss the structural aberrations and the related physicochemical properties of 2D MHEMs.

### 4.1 Severe Crystal Structure Distortion

**Figure 6:**
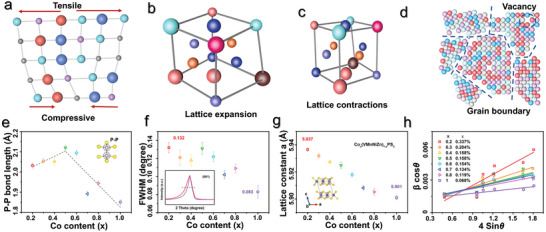
Structural aberrations of 2D MHEMs. a) Atom discrepancy induces structure compressive and tensile strain. b) Lattice expansion induced by tensile strain; c) Lattice contractions induced by compressive strain; d) Grain boundary and vacancy caused by severe lattice distortion. The crystal structure of 2D high‐entropy Co_x_(VMnNiZn)_1‐x_PS_3_ is regarded as an illustrative example. e) P─P bond length at different Co content. f) (001) plane full width at half‐maximum varies with Co content. g) The lattice constant a varies with Co content. h) Lattice strain ε calculated based on W‐H analysis. Adapted with permission.^[^ ^43^ ^]^ Copyright 2022, American Chemical Society.

The properties of 2D MHEMs largely depend on their phase structure and elemental composition. In contrast to traditional low‐entropy 2D materials with fixed bond length, 2D MHEMs usually exhibit tunable bond length and strength, induced by disordered atomic distributions and atomic sizes, which further affect the 2D microstructure. As shown in **Figure** **6**a–c, drastic‐lattice‐distortion effects of 2D MHEMs lead to the formation of tensile and compressive strain. In localized lattice, tensile strain induces lattice expansion, while compressive strain triggers lattice contraction. When the lattice strain is too strong for the 2D structure to withstand, the crystal structure will change dramatically, leading to the formation of grain boundary, phase separation, or polycrystalline structure (Figure 6d). For instance, Song et al. reported Rietveld refinement of XRD data to investigate the crystal structural aberration in high‐entropy Co_x_ (VMnNiZn)_1−x_PS_3_.^[^ ^43^ ^]^ As shown in Figure 6e, the internal P‐P bond length of Co_x_(VMnNiZn)_1−x_PS_3_ changed with Co content. Obviously, the original CoPS_3_ delivers a lower P‐P bond length than that of high‐entropy Co_x_(VMnNiZn)_1−x_PS_3_. Furthermore, the discrepancy of (001) full width at half‐maximum (FWHM) between CoPS_3_ and high‐entropy Co_x_(VMnNiZn)_1−x_PS_3_ also demonstrated lattice distortion (Figure 6f). At different Co contents, the lattice constant a of Co_x_(VMnNiZn)_1−x_PS_3_ was significantly different (Figure 6g). By comparison, high‐entropy Co_x_(VMnNiZn)_1−x_PS_3_ presented a larger lattice constant, indicating tensile structure along an axis. Meanwhile, the lattice strains of Co_x_(VMnNiZn)_1−x_PS_3_ were all calculated based on Williamson−Hall (W−H) analysis. As shown in Figure 6h, the lattice strain ε enhances gradually with the decrease of Co content. The in‐depth analysis of Co_x_(VMnNiZn)_1−x_PS_3_ lattice proved the structural aberrations induced by high‐entropy effects.

### 4.2 Abundant Defects and Atomically Lattice Strain

**Figure 7:**
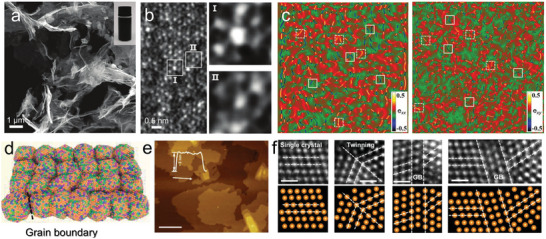
Structural features of 2D MHEMs. a,b) SEM and TEM images of high‐entropy (Ti_1/5_V_1/5_Zr_1/5_Nb_1/5_Ta_1/5_)_2_AlC derived MXene. Defects induced by lattice distortion are clearly observed. c) Strain distribution of high‐entropy MXene along e_xx_ and e_xy_ direction. A number of atomic lattice strains were detected. Reproduced with permission.^[^ ^64^ ^]^ Copyright 2021, Wiley‐VCH. d–f) Structure, AFM, and STEM images of 2D high‐entropy RulrFeCoNiO_2_ (scale bar: 2 µm for e, 0.5 nm for h). The RulrFeCoNiO_2_ delivers 3 nm thickness and abundant grain boundaries. Adapted under the terms of the CC‐BY‐NC licence.^[^ ^44^ ^]^ Copyright 2023, AAAS.

Similar to high‐entropy Co_x_(VMnNiZn)_1−x_PS_3_, structural aberrations were also inspected in other 2D MHEMs, such as high‐entropy (Ti_1/5_V_1/5_Zr_1/5_Nb_1/5_Ta_1/5_)_2_AlC MAX phase and the derived MXene.^[^ ^64^ ^]^ As shown in **Figure** **7**a, numerous transparent and twisted nanosheets were observed in TEM images. In addition, the microstructure of 2D high‐entropy MXene, including strong lattice mismatch and defects, was also revealed (Figure 7b). Furthermore, using atomic‐resolution high‐angle annular dark‐field images, geometric phase analysis was performed to demonstrate the distribution of lattice strain. As displayed in Figure 7c, compressive and tensile strain were both identified. Despite the strong lattice strain, the crystal structure and ultrathin nanosheet morphology was preserved. In addition to the discovery of lattice strain, the grain boundary was also detected in 2D MHEMs. For example, Huang et al. prepared misoriented high‐entropy RuIrFeCoNiO_2_, which exhibited uniform 2D morphology with 3 nm thickness and abundant grain boundaries (Figure 7d–f).^[^ ^44^ ^]^

### 4.3 Enhanced Structural Stability

**Figure 8:**
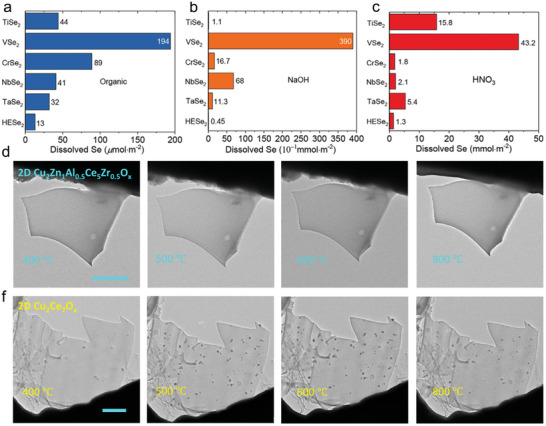
The strong structural stability of 2D MHEMs. a–c) Corrosion resistance comparison between high‐entropy (Ti, V, Cr, Nb, Ta)Se_2_ and parallel monocomponent under HNO_3_, NaOH, and organics conditions. Reproduced with permission.^[^ ^18^ ^]^ Copyright 2021, American Chemical Society. d–f) In situ TEM images of 2D high‐entropy Cu_2_Zn_1_Al_0.5_Ce_5_Zr_0.5_O_x_ and Cu_2_Ce_7_O_x_ for reverse water gas shift reaction in range of 400–800 °C. Reproduced under the terms of the CC‐BY‐NC licence.^[^ ^66^ ^]^ Copyright 2023, Springer Nature.

The entropy engineering could significantly affect the structural stability of 2D materials. The strong lattice distortion would create high energy barriers, slowing down atom diffusion, and resulting in superior thermal stability, mechanical strength, and corrosion stability. As shown in **Figure** **8**a–c, corrosion resistance of 2D high‐entropy (Ti, V, Cr, Nb, Ta)Se_2_ tests were conducted in HNO_3_ (8.1 m, 10 min), NaOH (0.5 m, 10 min) and n‐butylamine (1.0 mmol butylamine and 2 mL tetrahydrofuran mixing solution, 1 h).^[^ ^18^ ^]^ 2D (Ti, V, Cr, Nb, Ta)Se_2_ exhibited lower concentrations of dissolved Se than its counterparts, indicating enhanced corrosion resistance and higher stability. In practical applications, the irreversible leaching and corrosion of metal active sites remain still challenging. Shao et al. reported the high‐entropy effect confers 2D FeCoMoWO_x_ self‐healing behavior in oxygen evolution reaction (OER).^[^ ^65^ ^]^ In a borate buffer containing cobalt ions condition, 2D FeCoMoWO_x_ shows an overpotential reduction behavior and long‐term stability of 100 h in catalysis. In addition to the corrosion resistance, 2D MHEM also present higher thermal stability (Figure 8d–f), making them ideal candidates for applications in harsh conditions. For instance, as a catalyst for reverse water gas shift reaction (RWGS), 2D high‐entropy Cu_2_Zn_1_Al_0.5_Ce_5_Zr_0.5_O_x_ retains the morphological structure even at 800 °C.^[^ ^66^ ^]^ In contrast, nanoparticle sintering was observed in the Cu_2_Ce_7_O_x_ catalyst during the heating process at lower temperatures, suggesting poor thermostability. Thus, the structural stability of 2D MHEMs can be exploited for catalytic reactions under harsh conditions.

### 4.4 Versatile Electromagnetic Properties

**Figure 9:**
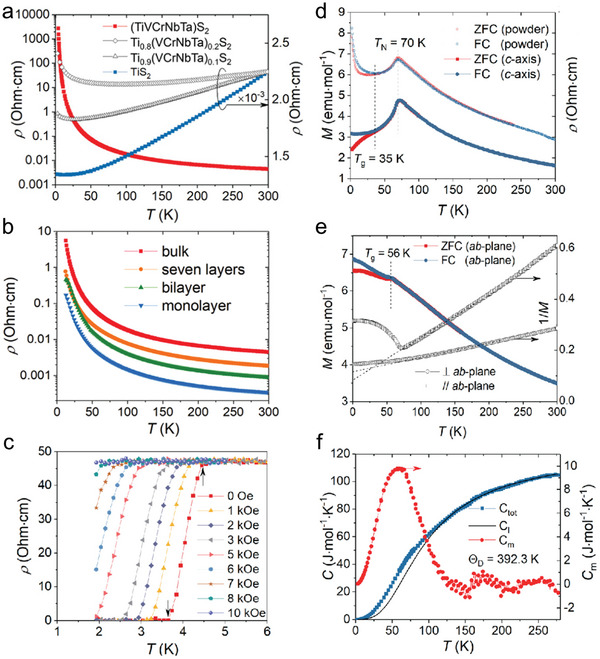
Unique properties of 2D MHEMs, including electrical and magnetic properties. a) Resistivity curves. The Metal‐insulator transition was influenced by composition in high‐entropy (TiVCrNbTa)S_2_. b) Resistivity of high‐entropy (TiVCrNbTa)S_2_ at different thickness from 12 to 300 K. c) Superconductivity of high‐entropy (Co, Au)_0.2_(Rh,Ir,Pt,Pd)_0.8_Te_2_. d,e) The magnetization tests of (Mn,Fe,Co,Ni)PS_3_, which were conducted under the external magnetic field H = 500 Oe vertical to or parallel the ab‐plane. f) Heat capacity of (Mn,Fe,Co,Ni)PS_3_ from 2 to 280 K. Reproduced with permission.^[^ ^18^ ^]^ Copyright 2021, American Chemical Society.

The unique structural versatility and chemical diversity of 2D MHEMs offer a flexible platform for discovering unconventional physical and chemical phenomena. For instance, Hideo et al. systematically investigated the physical characteristics, including electrical and magnetic properties of high‐entropy vdW materials (**Figure** **9**), and their relationship with chemical tunability.^[^ ^18^ ^]^ As shown in Figure 9a, a metal‐insulator transition phenomenon was observed between (TiVCrNbTa)S_2_ and original TiS_2_ due to high entropy effects. Furthermore, in contrast to the electrical behavior of conventional bulk TMC, the resistivity of high‐entropy (TiVCrNbTa)S_2_ witnessed a decrease upon thickness reduction (Figure 9b). Surprisingly, superconductivity was also observed in high‐entropy (Co,Au)_0.2_(Rh,Ir,Pt,Pd)_0.8_Te_2_, which might be ascribed to the formation of Te‐Te dimer (Figure 9c). The magnetic properties of high‐entropy vdW materials were also explored. For instance, pristine mono‐metal phosphorus trichalcogenides (i.e., (Mn,Fe,Co,Ni)PS_3_) exhibit characteristic behaviors of Heisenberg magnets and anisotropic Heisenberg magnets in MnPS_3_ and NiPS_3_, respectively. As shown in Figure 9d, (Mn,Fe,Co,Ni)PS_3_ presents an antiferromagnetic transition behavior at 70 K under an external magnetic field along the *c*‐axis. As the temperature dropped, a spin glass transition was also observed at 35 K. Once setting an external magnetic field along the ab‐plane direction, spin glass transition appeared at 56 K (Figure 9e). The heat capacity test further confirmed the Heisenberg‐type antiferromagnetic transition (Figure 9f). So far, the research on electrical and magnetic properties of 2D MHEM is still at a preliminary stage. Therefore, considering the complexity of 2D MHEM structure and composition, further efforts are needed to find the influencing factors such as atomic radius, electronegativity, chemical bond length, lattice distortion, and thickness. High‐throughput computation might be a useful tool to predict the structure‐to‐properties relationship by constructing accurate atomic distribution and electronic structure.

## 5 2D MHEMs Applications

### 5.1 Catalysis

The development of clean, renewable, and affordable energy technologies can reduce the extensive use of fossil fuels, which is crucial for the global transition to a greener, more sustainable future. Considering the highly abundant presence in nature, carbon dioxide and water serve as potential sources for generating efficient energy carriers like hydrogen, methanol, and ethanol.^[^ ^67^ ^]^ This objective can be achieved through techniques like photocatalysis, thermocatalysis, and electrocatalysis, including chemical reactions such as hydrogen evolution reaction (HER), oxygen evolution reaction, oxygen reduction reaction (ORR), carbon dioxide reduction reaction (CO_2_RR), and others.^[^ ^68^ ^]^ To date, the majority of catalysts are composed of precious metals such as palladium, platinum, and ruthenium, thus they are impractical for industrial‐scale production due to their scarcity and high cost.^[^ ^69^ ^]^ As a result, the development of high‐performance catalysts through the substitution of precious metals with high‐abundance elements is highly sought after.

**Figure 10:**
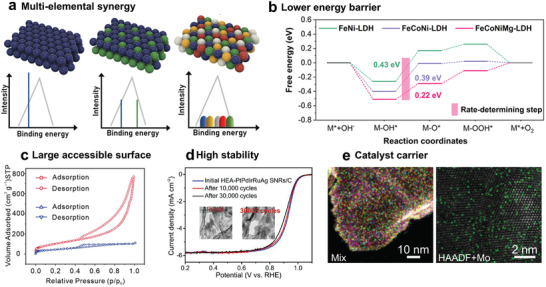
a) The multi‐elemental synergy mechanism of high‐entropy MHEMs. The diverse element types, concentration, and unordered distribution of MHEMs contribute to continuous surface binding energy. Reproduced with permission.^[^ ^70^ ^]^ Copyright 2021, Wiley‐VCH. b) The variations comparison of Gibbs free energy in the four‐step OER process. It is apparent that FeCoNiMg‐LDH delivers more favorable catalysis kinetics. Reproduced with permission.^[^ ^71^ ^]^ Copyright 2023, American Chemical Society. c) N_2_ adsorption and desorption curves of MOFs derived monolayer high‐entropy hydrotalcites (red) and amorphous hydrotalcites obtained by the alkali coprecipitation method (blue). The calculated specific surface area is 380.5 and 164.2 mg^2^/g. Reproduced with permission.^[^ ^52^ ^]^ Copyright 2023, Wiley‐VCH. d) Linear sweep voltammetry curves and corresponding TEM images of high‐entropy PtPdIrRuAg alloy nanoribbons during cycling. Reproduced with permission.^[^ ^38^ ^]^ Copyright 2022, American Chemical Society. e) Mo single atoms dispersed in 2D high‐entropy PdPtNiCuZn alloys. Reproduced under the terms of the CC‐BY‐NC licence.^[^ ^78^ ^]^ Copyright 2024, Springer Nature.

2D MHEMs, in view of their high chemical tunability, multi‐metal sites, and large surface area, are potential alternative catalysts. **Table** **2** summarizes the catalytic performances of representative 2D MHEMs and corresponding counterparts. Obviously, 2D MHEMs show excellent catalytic activity and high stability in a variety of catalytic reactions, superior to low‐entropy or bulk forms. In contrast to the discrete nature of binding energies in traditional materials, 2D MHEMs with unordered element distribution present uniform and wideband binding energies, which is the key to superior performance (**Figure** **10**a).^[^ ^70^ ^]^ Thus, the excellent catalytic performance does not only arise from the elemental nature of MHEMs, but also from the synergistic induced effects, namely cocktail effects, which play a dominant role. Considering the high tunability in element types and concentrations, there are ample opportunities to integrate diverse active sites to optimize binding energy and achieve enhanced catalytic performances. Furthermore, the synergistic working effects can be evaluated by computational chemical methods. For example, in the four‐step OER pathway under alkaline conditions, the electronic structure and variations of Gibbs free energy can be estimated by density functional theory calculations. As shown in Figure 10b, FeCoNiMg‐LDH displays a lower energy barrier than FeNi‐LDH and FeCoNi‐LDH in the rate‐determining step.^[^ ^71^ ^]^ The high‐entropy coordination of FeCoNiMg‐LDH generates synergistic electronic interactions that activate nickel (Ni) in spin‐polarized states, elevating the Fermi level. This, in turn, optimizes the adsorption energy of intermediates and speeds up the kinetics of the oxygen evolution reaction (OER). Besides, the synthesis of FeCoNiMg‐LDH, which incorporates elevated configuration entropy and reduced Gibbs free energy, coupled with the formation of Mg─O interfacial bonds, ensures a stable crystal structure and mitigates phase segregation, enabling exceptional stability and long‐term catalytic performance.

**Table 2:** Catalytic performance of representative 2D MHEMs.

HER	Electrolyte	Overpotential [mV]	Tafel scope [mV dec^−1^]	Stability	Ref
PdMoGaInNi alloy	0.5 m H_2_SO_4_	13	149	12 h no decay	[73]
Pd		42	185.1	Unstable	
Co_0.6_(VMnNiZn)_0.4_PS_3_	1 m KOH	65.9	65.5	12 h small loss	[43]
CoPS_3_		202.8	93.5	∼	
PdPtRhlrCu	1 m KOH	15	37	20 h small loss	[79]
Pd		273	173	∼	
(MoWReMnCr)S_2_	0.5 m H_2_SO_4_	127	111.6	20 h small loss	[55]
MoS_2_		219	133.8	∼	
OER	Electrolyte	Overpotential [mV]	Tafel scope [mV dec^−1^]	Stability	
LiMoFeCoNi‐LDH	1 m KOH	187	82	12 h no decay	[46]
FeNi‐LDH		232	112	∼	
M‐RulrFeCoNiO_2_	0.5 m H_2_SO_4_	189	49	120 h no decay	[44]
M‐RuO_2_		210	51	10 h large loss	
FeCoMoWO_x_	1 m KOH	332	63.6	self‐healing	[65]
FeCoOx		365	63.2	self‐healing	
(NiFeCoMn)_3_S_4_	1 m KOH	289	75.6	1000 cycles no decay	[80]
(NiFe)_3_S_4_		413	101.6	∼	
GaFeCoNiMo	1 m KOH	240	37.9	250 h small loss	[63]
GaFeCoNi		383	60.6	∼	
ZnCoNiFeV‐LDH	1 m KOH	253	49	1000 h small loss	[52]
NiCo‐LDH		347	∼	∼	
FeCoNiMg‐LDH	1 m KOH	302	75	60 h small loss	[71]
FeNi‐LDH		347	83	60 h large loss	
Au_SA_‐MnFeCoNiCuLDH	1 m KOH	213	27.5	700 h small loss	[42]
MnFeCoNiCuLDH		323	85.5	∼	
ORR	Electrolyte	Half‐wave potential [V]	Mass activity [A mg noble metal^−1^]	Stability	
PtPdlrRuAg alloy	0.1 m KOH	0.93	4.28	10 000 cycles	[38]
Pt/C		0.85	0.2	Unstable	
CoFeNiMnCuZnO_x_‐PMA	0.1 m KOH	0.802 under light	∼	∼	[81]
NiMnCuZnO_x_‐PMA		∼0.700 under light	∼	∼	
Pt_32_Pd_48_Ni_20_	0.1 m KOH	0.89	0.54	10 000 cycles no decay	[37]
Pd/C		0.82	0.07	∼	
CO_2_RR	Electrolyte	Current (A cm^−2^)	Potential (V)	Stability	
(MoWNbTaV)S_2_	1 m KOH and 1 m choline chloride	0.51	−0.8	20 h small loss	[75]
RWGS	Feed gas	CO production rate (mmol g^−1^ h^−1^)	Selectivity	Stability	
Cu_2_Zn_1_Al_0.5_Ce_5_Zr_0.5_O_x_	CO_2_/H_2_ = 1/1 with 40 sccm flow rate	417.2	100%	stable	[66]
Cu_2_Ce_7_O_X_		∼50	∼	unstable	

Considering that most of the catalytic reactions occur at interfaces, the surface structure of the catalyst is key. In general, nanoscale electrocatalysts deliver enhanced catalytic properties than bulk counterparts due to the greater amount of exposed metal sites.^[^ ^72^ ^]^ In this case, the atomic‐thickness effects endow MHEMs with a large accessible surface area. For instance, MOF‐derived monolayer high‐entropy hydrotalcites exhibit a large specific surface area of 380.5 m^2^ g^−1^, which promotes the catalytic efficiency (Figure 10c).^[^ ^52^ ^]^ In addition, another key point worth mentioning is the sluggish diffusion effects of 2D MHEMs. Several studies have demonstrated that the sluggish diffusion effects slow down the phase transition and stabilize the microstructure of MHEMs, which contributes to high stability in catalytic reactions.^[^ ^73^, ^74^, ^75^, ^76^ ^]^ As shown in Figure 10d, even after 30 000 cycles, high‐entropy PtPdIrRuAg alloy nanoribbons still remain similar catalytic performance and morphologic structure in ORR.^[^ ^38^ ^]^ Especially, the high durability of 2D MHEMs greatly increases their potential in harsh environments. For instance, in seawater electrolysis, the catalyst must endure oxidation and reduction induced by the electric field, as well as withstand corrosion from seawater.^[^ ^77^ ^]^ Despite this, the loss of catalytic activity is still inevitable in long‐term work under harsh conditions. So, this raises the question: Can the activity of the catalyst be regenerated? In a recent work by Kang et al., self‐healing of catalytic activity was discovered in 2D high‐entropy oxides.^[^ ^65^ ^]^ After 100 h cycling in borate buffer (KBi, pH = 14) containing Co^2+^ electrolyte solution, the FeCoNiWO_x_ catalyst shows reduce of overpotential and Tafel slope. However, the underlying mechanism of self‐healing still needs further investigation. In situ characterizations during electrocatalytic reactions might enhance the understanding of the evolution of catalyst structures during self‐healing. Recently, 2D MHEMs have also been used as catalyst carriers to design high‐performance compound catalysts. The atomic‐thickness effect endures ample surface area for loading various highly active atoms.^[^ ^42^ *^,^* ^78^ ^]^ For instance, single‐atom Mo was shown to be deposited in 2D high‐entropy PdPtNiCuZn alloys to catalytic methanol oxidation reactions. This offers a novel approach to designing high‐performance catalysts, namely the precise structural integration of 2D MHEMs with other high‐activity components (Figure 10e).

### 5.2 Energy Storage

Developing renewable and clean energy sources to replace the burning of fossil fuels, such as wind, tidal, and solar energy, is instrumental in tackling the escalating environmental issues and global climate changes.^[^ ^82^, ^83^, ^84^ ^]^ Electrochemical energy storage technology, as a key supporting technology for the energy revolution, plays a decisive role in advancing society. To date, much attention has been paid to the development of various electrochemical energy storage devices, including supercapacitors, lithium‐ion batteries (LIB), potassium‐ion batteries (PIB), lithium metal batteries (LMB), and lithium‐sulfur batteries.^[^ ^85^, ^86^, ^87^ ^]^ Despite the progress, electrochemical energy storage devices still rely on conventional architectures based on cathode, anode, electrolyte, and separator, respectively.^[^ ^88^ ^]^ Thus, it is expected that high‐performance devices can be achieved through the utilization of advanced materials in the construction of these components.

**Figure 11:**
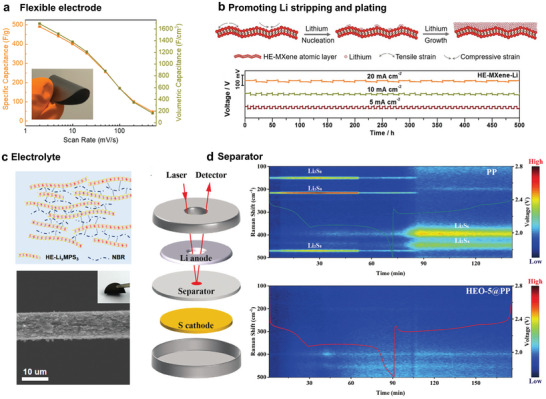
Energy storage applications of 2D MHEMs. a) 2D high‐entropy Ti_1.1_V_0.7_Cr_x_Nb_1.0_Ta_0.6_C_3_T_z_ as flexible electrode for supercapacitors. Obviously, MXene is easily integrated into flexible membrane electrodes via vacuum filtration. Reproduced with permission.^[^ ^40^ ^]^ Copyright 2022, American Chemical Society. b) Li stripping and plating on (TiVZrNbTa)_2_CT_x_ surface. 2D (TiVZrNbTa)_2_CT_x_ is stable in 500 h galvanostatic cycling. Reproduced with permission.^[^ ^64^ ^]^ Copyright 2021, Wiley‐VCH. c) 2D high‐entropy Li_x_(Fe_1/5_Co_1/5_Ni_1/5_Mn_1/5_Zn_1/5_)PS_3_ with high Li^+^ conductivity was applied to build solid‐state electrolytes in lithium metal batteries. Reproduced with permission.^[^ ^91^ ^]^ Copyright 2023, American Chemical Society. d) 2D high‐entropy LaFe_0.4_Co_0.2_Ni_0.2_Cu_0.2_O_3_ was applied to construct the Li‐S batteries separator. In situ Raman spectrums prove the suppression effect of LaFe_0.4_Co_0.2_Ni_0.2_Cu_0.2_O_3_ on lithium sulfide precipitation. Reproduced with permission.^[^ ^92^ ^]^ Copyright 2024, Wiley‐VCH.

Among various electrochemical energy devices, supercapacitors and LIB attracted the most attention. Supercapacitors offer the advantages of high‐power density (>1000 W kg^−1^) and stable cycling life (over 10 000 cycles), but they suffer from low energy density (<10 Wh/kg).^[^ ^89^ ^]^ On the contrary, LIB presents favorable energy density, but ordinary power density and stability. In this case, the 2D MHEMs with high chemical tunability are the potential option to solve these fundamental challenges. **Table** **3** reviews the energy storage performances of representative 2D MHEMs. Generally, there are two main electrochemical mechanisms for both supercapacitors and batteries: capacitive contribution related to the surface structure and faradaic pseudocapacitance contribution involved with elements' redox properties.^[^ ^90^ ^]^ Compared to conventional materials, 2D MHEMs electrodes feature superior capacity, favorable rate capability, and long cycling life, which is ascribed to its core effects. First, the atomic thickness of 2D MHEMs guarantees optimal interface, through enhanced contact, with the surface of the electrode and the electrolyte, which is the basis of an efficient charge transfer between electrolyte and electrode. Moreover, the ultimate thinness extended the application of MHEMs to flexible energy storage devices. As shown in **Figure** **11**a, 2D high‐entropy Ti_1.1_V_0.7_Cr_x_Nb_1.0_Ta_0.6_C_3_T_z_ is easily integrated into a flexible membrane electrode via vacuum filtration.^[^ ^40^ ^]^ Second, the strong lattice distortion of 2D MHEMs improves the kinetics of redox reactions at the electrode/electrolyte interface, leading to increased capacity. Last, 2D MHEMs show high durability being key to suppressing the occurrence of side reactions during cycling. In this context, Yang et al. showed that high mechanical strain induced by lattice distortion could effectively promote the Li nucleation and growth uniformly on the high‐entropy (TiVZrNbTa)_2_CT_x_ surface (Figure 11b), resolving the dendritic crystal issue.^[^ ^64^ ^]^ Furthermore, stable stripping and plating of Li can last up to 500 h in a galvanostatic cycling test, indicating favorable stability. Due to their chemical and structural versatility, 2D MHEMs can also be employed as electrolytes and separators. As shown in Figure 11c, Yang et al. discovered that 2D high‐entropy Li_x_(Fe_1/5_Co_1/5_Ni_1/5_Mn_1/5_Zn_1/5_)PS_3_ serves as an excellent Li^+^ conductor, boasting high conductivity up to 5 × 10^−4^ S cm^−1^.^[^ ^91^ ^]^ To verify the practical application capability, Li_x_(Fe_1/5_Co_1/5_Ni_1/5_Mn_1/5_Zn_1/5_)PS_3_ was integrated with nitrile‐butadiene rubber to construct a solid‐state electrolyte for lithium metal batteries. Similarity, 2D MHEMs can also be assembled into a separator to catalytic the S conversion in Li‐S batteries (Figure 11d).^[^ ^92^ ^]^ The in situ Raman spectrums reveal that LaFe_0.4_Co_0.2_Ni_0.2_Cu_0.2_O_3_ based separator suppresses the precipitation of lithium sulfides, which contributes to a high capacity and stable cycling life. Overall, the core effects of 2D MHEMs have shown considerable impacts on electrochemical performances yet their potential in advanced energy storage applications is still uncharted.

**Table 3:** Energy storage performance of representative 2D MHEMs.

Supercapacitors	Capacity [F/g]	Rate capability	Stability	Ref
Ti_2_V_0.9_Cr_0.1_C_2_T_x_	553.3	good	small loss after 2000 cycles	[41]
Ti_3_C_2_T_x_	∼350	bad	∼	
Ti_1.1_V_0.7_Cr_x_Nb_1.0_Ta_0.6_C_3_T_z_	490	good	89% retention after 9000 cycles	[40]
LIB	Capacity [mAh/g]	Rate capability	Stability	
(TiVNbTaMo)₂C	283	good	300 cycles no decay	[93]
(TiVNbTa)_2_C	276	bad	300 cycles no decay	
(TiVNb)_2_C	276	moderate	300 cycles no decay	
(CoCuMgNiZn)O	450	good	200 cycles no decay	[47]
PIB	Capacity [mAh/g]	Rate capability	Stability	
(CoVMnFeZn)PS_3_	524	good	1000 cycles no change	[94]
(CoMnFe)PS_3_	≈520	moderate	400 cycles large loss	
FePS_3_	≈480	bad	200 cycles large loss	
LMB solid electrolyte	Li^+^ conductivity [S cm^−1^]	Coulombic efficiency	Stability	
Li_x_(Fe_1/5_Co_1/5_Ni_1/5_Mn_1/5_Zn_1/5_)PS_3_	5 × 10^−4^	99.8%	800 h small loss	[91]
Li_x_FePS_3_	≈1 × 10^−6^	∼	∼	
LMB	Li nucleation overpotential [mV]	Rate capability	Stability	
(TiVZrNbTa)_2_CT_X_	6	good	small loss after 1200 h	[64]
Ti_2_C_2_T_x_	34	bad	large loss after 500 h	
Li‐S separator	Catalyze sulfur conversion	Capacity [mAh/g]	Stability	
(TiVZrNbTa)_2_C_x_N_1–x_T_y_	efficient	702	small loss after 360 cycles	[95]
Ti_2_C_1/2_N_1/2_T_x_	inefficient	∼	∼	
TiVNbMoC_3_	efficient	948.5	1200 cycles no decay	[96]
Ti_4_C_3_	inefficient	∼	∼	
LaFe_0.4_Co_0.2_Ni_0.2_Cu_0.2_O_3_	efficient	1199.8	0.041% decay per cycle	[92]
SERS	Bandgap [eV]	Detection limit [R6G]	Stability	
MnFeCuAgInPS_3_	0.43	10^−9^ m	6 months, workable in acid/alkaline conditions	[97]
MnPS_3_	2.35	Bad	∼	

### 5.3 Electronic Applications

**Figure 12:**
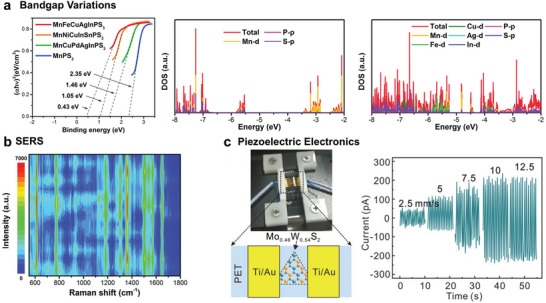
a) Bandgap variations induced by entropy engineering toward transition‐metal phosphorus sulfides. The band gap spectra of MnPS_3_ MnCuPdAgInPS_3_, MnFeCuAgInPS_3_ and MnNiCuInSnPS_3_ are presented. Density functional theory calculations were performed and the density of the state of Mn_8_P_8_S_24_ (MnPS_3_ model) and Mn_2_Fe_2_Cu_2_Ag_1_In_1_P_8_S_24_ (MnNiCuInSnPS_3_ model) are displayed. b) SERS performance of rhodamine 6G (10^−6^ M) on MnNiCuInSnPS_3_. Reproduced with permission.^[^ ^97^ ^]^ Copyright 2024, Wiley‐VCH. c) Piezoelectric application of 2D medium‐entropy Mo_0.46_W_0.54_S_2_. Reproduced with permission.^[^ ^104^ ^]^ Copyright 2022, Wiley‐VCH.

The extended compositional and structural tunability of 2D MHEMs can be further exploited to broaden the range of applicability, to include electronic devices application.^[^ ^98^, ^99^, ^100^ ^]^ As the progress in electronics is still at an early stage, understanding how entropy engineering can alter the electronic/magnetic/thermal properties represents a core issue.^[^ ^101^ ^]^ For instance, the strong lattice distortion in semiconducting 2D MHEMs induces a series of structural defects, such as vacancies and grain boundaries, which directly affect the carrier migration, making the design of 2D MMHEs with tailored semiconducting characteristics represent a major challenge. Nevertheless, preliminary studies in the development of semiconductive 2D MHEMs by tuning their composition to modulate the band structure have been carried out.^[^ ^102^ ^]^ As shown in **Figure** **12**a, the spectral and Mott–Schottky characterizations revealed that entropy engineering of 2D MnPS_3_ effectively changes valence and conduction band, thus acquiring high‐entropy MnNiCuInSnPS_3_ with 0.43 eV bandgap.^[^ ^97^ ^]^ To gain insight into the influence of each element on band structure, DFT calculations were performed on an optimized crystal structure model. The substantial continuous *d*‐electron states of Mn, Fe, and Cu play a predominant role in filling and narrowing the bandgap instead of the *p*‐electron states of P and S atoms (Figure 12a). This facilitates the occupation of energies near the Fermi level and increases the density of states, which suggests the regulation of electronic structure from component modulation. To explore practical applications, 2D high‐entropy MnNiCuInSnPS_3_ was employed in surface‐enhanced Raman scattering, exhibiting a low detection limit with 10^−9^ M for rhodamine 6G.

2D MHEMs also show potential in other electronics applications. Entropy engineering has been demonstrated to promote the enhanced capacitive energy storage of dielectrics.^[^ ^103^ ^]^ In the latest work, an entropy‐stabilized dielectric film based on Bi_2_Ti_2_O_7_ was reported and boasting an impressive energy density of 182 J cm^−3^ with an efficiency of 78% at 6.35 MV cm^−1^. In‐depth studies indicate that favorable and stable microscopic structural characteristics are introduced upon modulation of the atomic configuration entropy, including lattice‐distorted nanocrystals and disordered quasi‐amorphous phases, which deliver enhanced disruptive strength, energy storage density, and efficiency. Moreover, entropy engineering was also adopted to improve the piezoelectric and thermoelectric performance of transition metal chalcogenide.^[^ ^104^ ^]^ For example, a piezoelectric coefficient of 4.22 pm V^−1^ was achieved in 2D medium‐entropy Mo_0.46_W_0.54_S_2_, much higher than that of bare MoS_2_ and WS_2_ (Figure 12c). Overall, the electronic applications of 2D MHEMs are still in the early stages, and more effects are needed to reveal the relationship between the core effects of MHEMs and electronic/magnetic/thermal properties.

**Figure 13:**
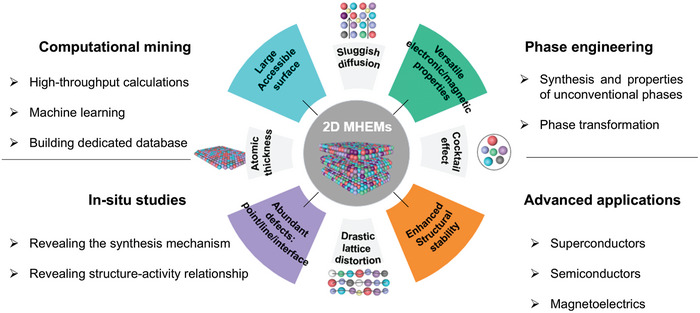
Schematic depiction of the core effects, properties, and future developments of 2D MHEMs.

Understanding the structure‐to‐activity relationship in 2D MHEMs is critical to exploit their full potential. 2D MHEMs present core effects, including entropy, lattice distortion, sluggish diffusion, cocktail, and atomic thickness, as shown in **Figure** **13**. In addition, some MHEMs exhibit further characteristic behaviors that differ from mono‐ and bi‐elemental 2D systems. First, 2D MHEMs show more accessible and larger active surfaces than conventional materials, enhancing the catalytic performance. Second, due to the diversity of atoms involved, pronounced lattice distortion of 2D MHEMs induces strong mechanical strain, enabling unusual curved nanosheet morphology, inducing the increase of active surface and endowing deformability function. Third, 2D MHEMs present abundant defects, lattice distortions, grain boundaries, and unsaturated sites, changing the intrinsic electronic structure and chemical activity. For instance, enhanced electrocatalytic activity and sluggish dynamics in energy storage devices were observed because of the crystal defects and vacancies.

## 6 Summary and Outlook

The combination of entropy engineering and atomic thickness has delivered a vast and versatile library of 2D materials with enticing physicochemical properties. So far, a series of 2D MHEMs have been designed and produced, such as MXene, oxides, TMC, LDH, and alloys. Thus, to gain insights into this emerging class of materials, we critically assessed their synthesis, structure, properties, and applications. (Figure 13) First, state‐of‐the‐art synthetic techniques, including wet‐chemical doping, CVD doping, and atomic exchange alloying were discussed, bringing forward the need for further efforts toward the achievement of phase control in 2D MHEMs. Second, structural defects (i.e., vacancies, dislocations, unsaturated sites, and grain boundaries) and atomically distributed lattice strains arising from the lattice distortion induced by the 2D confinement were reviewed, as they strongly affect the thermal, electrical, and magnetic properties of 2D MHEMs and thus, their applications. In particular, the core effects of 2D MHEMs, such as cocktail, sluggish‐diffusion, atomic‐thickness, and drastic‐lattice‐distortion, play critical roles in the device performance of specific applications ranging from catalysis to (opto)electronics and energy storage. Despite of great progress achieved thus far, some key points require more attention for the future endeavor in this research direction.

### 6.1 Computational Mining

To date, the experimental approaches driven by trial‐and‐error strategies limit the advancement of 2D MHEMs. In contrast, due to the structural complexity and diversity of this class of materials, the integration of computational strategies could be instrumental in boosting the exploration and prediction of novel architectures. High‐throughput calculations and machine learning can be exploited as powerful strategies to predict the fundamental properties of uncharted 2D MHEMs. For example, the calculated phase diagram method (CALPHAD) has been employed to identify the mechanical properties of high‐entropy alloys.^[^ ^105^ ^]^ Nevertheless, the structural complexity as well as the uncertainty of atomic arrangement call for high computational power. Current calculations mainly focus on the thermodynamic equilibrium system of the bulk materials and have not yet been expanded to the 2D regime. Additionally, existing materials databases, such as *Computational 2D Materials Database (C2DB), Materials Cloud*, and *Materials Project*, contain limited examples of MHEMs that are clearly described or labeled, failing to offer enough statistics for machine training algorithms. Therefore, establishing a dedicated database for such materials would foster their development.

### 6.2 Phase Engineering

The structural phase shaped by the arrangement and organization of atoms within the lattice is crucial to impart 2D MHEMs programmed mechanical, thermal, and electrical properties. However, the precise control over the structural phase of 2D MHEMs represents still a challenge. Indeed, phase engineering in MHEMs is hampered by the endless combination of the elemental composition induced by extreme synthesis conditions in the state‐of‐the‐art approaches. Thus, developing growth techniques with atomic precision would represent a step forward toward phase control of these architectures. Currently, 2D MHEMs are restricted to a reduced amount of phase structures, such as MXene, hydrotalcites, and alloy with Face Center Cubic (FCC) structure. Other forms including 2D nitrides and phosphide are still unexplored as efficient synthetic methodologies are still missing. Designing multiphase 2D MHEMs‐based composites or heterostructures, especially unconventional crystal phases, represents an avenue holding great promise in fundamental science and practical applications, as new physicochemical properties of 2D MHEMs can be uncovered.

### 6.3 In Situ Studies

Further efforts are required to unveil the structure versus property relationship in such a wide family of materials. Advanced characterizations have played a pivotal role in thoroughly assessing the complex chemical composition of 2D MHEMs. So far, the phase, morphology, and elemental composition of 2D MHEMs have been explored by advanced structural characterizations like diffraction (X‐ray and neutron diffraction), microscopy (AFM, SEM, TEM, STEM), and spectroscopy (XPS, XANES, EXAFS) techniques. To retrieve correlations between synthesis and fundamental structural information of 2D MHEMs, in situ characterizations need further exploration. First, in situ and *operando* characterizations can monitor the synthetic reaction process cast light onto key parameters of the growth mechanism, and target the design of MHEMs with desired structures. For example, a high‐temperature alloying reaction was mimicked in *operando* environmental TEM, which enabled the monitoring of the fusion‐splitting‐fission‐crystallization growth process in high‐entropy alloy nanoparticles on liquid Ga.^[^ ^62^ ^]^ Moreover, in situ and *operando* characterizations can be exploited to provide further insights into the structural evolution of MHEMs using on‐chip electronic devices under operational conditions. For instance, the lattice distortion evolution of 2D MEHMs during the catalytic process, as well as the intercalation and de‐intercalation of ions occurring in the 2D MHEMs lattice in batteries, can be elucidated. Therefore, dynamic structural characterizations are of paramount importance in unraveling the structure versus properties relationship of 2D MHEMs, ultimately boosting their technological relevance.

### 6.4 Advanced Applications

The large compositional and structural diversity of 2D MHEMs provides a versatile platform for a wide range of applications. The majority of reported works have delivered successful proof‐of‐concept devices in the field of electrocatalysis (i.e., HER, OER, ORR, and CO_2_RR) and energy storage (i.e., supercapacitors and batteries). To further extend the scope of reach, combinatorial synthesis, controlling the elemental composition of 2D MHEMs, can be used to replace conventional toxic or noble metals as catalysts, achieving high catalytic activity and stability at reduced costs and detrimental environmental impact. In addition, 2D MHEMs represent a valuable alternative as electrode materials for solving the capacity loss caused by surface reconstruction and phase transition during the rapid charge‐discharge process or long‐term cycles occurring in energy storage devices. Also, enhanced stability can be achieved through compositional engineering, providing stable electrode materials for applications under extreme conditions, such as high temperature and high pressure. To push forward the development of such a versatile class of emerging materials, the 2D design and entropic engineering, leading to the control over the phase and electronic structure, will enable the exploration of multifunctional devices in the fields of optoelectronics, photonics, sensing, and beyond.

Ultimately, the 2D MHEMs are key components with chemically programmable and enhanced functional complexity that can be exploited in future disrputive high performance applications in catalysis, energy storage, and electronics, thereby becoming gamechangers for addressing global challenges.

## Conflict of Interest

The authors declare no conflict of interest.
